# MiR-378a-3p Acts as a Tumor Suppressor in Colorectal Cancer Stem-Like Cells and Affects the Expression of MALAT1 and NEAT1 lncRNAs

**DOI:** 10.3389/fonc.2022.867886

**Published:** 2022-06-24

**Authors:** Giorgia Castellani, Mariachiara Buccarelli, Valentina Lulli, Ramona Ilari, Gabriele De Luca, Francesca Pedini, Alessandra Boe, Nadia Felli, Mauro Biffoni, Emanuela Pilozzi, Giovanna Marziali, Lucia Ricci-Vitiani

**Affiliations:** ^1^Department of Oncology and Molecular Medicine, Istituto Superiore di Sanità, Rome, Italy; ^2^Core Facilities, Istituto Superiore di Sanità, Rome, Italy; ^3^Department of Clinical and Molecular Medicine, UOC Anatomia Patologica, Sant’Andrea Hospital, Sapienza University of Rome, Rome, Italy

**Keywords:** MiR-378a-3p, lncRNAs, MALAT1, NEAT1, colorectal cancer, colorectal cancer stem-like cells

## Abstract

MiR-378a-3p plays a critical role in carcinogenesis acting as a tumor suppressor, promoting apoptosis and cell cycle arrest and reducing invasion and drug resistance in several human cancers, including colorectal cancer (CRC), where its expression is significantly associated with histological classification and prognosis. In this study, we investigated the biological and cellular processes affected by miR-378a-3p in the context of CRC carcinogenesis. In agreement with the literature, miR-378a-3p is downregulated in our cohort of CRC patients as well as, in 15 patient-derived colorectal cancer stem-like cell (CRC-SC) lines and 8 CRC cell lines, compared to normal mucosae. Restoration of miR-378a-3p restrains tumorigenic properties of CRC and CRC-SC lines, as well as, significantly reduces tumor growth in two CRC-SC xenograft mouse models. We reported that miR-378a-3p modulates the expression of the lncRNAs MALAT1 and NEAT1. Their expression is inversely correlated with that of miR-378a-3p in patient-derived CRC-SC lines. Silencing of miR-378a-3p targets, MALAT1 and NEAT1, significantly impairs tumorigenic properties of CRC-SCs, supporting the critical role of miR-378a-3p in CRC carcinogenesis as a tumor-suppressor factor by establishing a finely tuned crosstalk with lncRNAs MALAT1 and NEAT1.

## Introduction

Colorectal cancer (CRC) is the third most diagnosed form of cancer in adults and the second most fatal tumor worldwide, with about 881,000 deaths estimated per year (1). CRC development is a multistep and complex process and results from the sequential accumulation of genetic and epigenetic alterations in several oncogenes and tumor suppressor genes (i.e., APC, KRAS, and TP53) leading to the transformation of colorectal epithelial cells to invasive adenocarcinomas (2). Currently, the standard chemotherapy regimens for advanced and metastatic CRC are the combination of a fluoropyrimidine with oxaliplatin (FOLFOX) and/or irinotecan (FOLFIRI) (3, 4).

Standard chemotherapies target proliferating cells, representing the majority of the tumor cell population, but they are not able to eliminate the highly tumorigenic subpopulation of colorectal cancer stem-like cells (CRC-SCs) (5). Due to their intrinsic self-renewing and differentiating potential, CRC-SCs are responsible for tumor initiation, progression and relapse (5, 6). Moreover, these cancer cells have a great ability to resist or to adapt to standard treatments through several mechanisms, leading to therapeutic resistance (7). Thus, the molecular characterization of CRC-SCs is fundamental for the development of new therapeutic strategies that would specifically target this subpopulation of cancer cells and increase the efficacy.

Several studies report the correlation between dysregulated non coding RNAs (ncRNAs) and the anomalous regulation of signaling pathways involved in CRC initiation and progression (8). NcRNAs are separated into two major categories, broadly based on their size: long ncRNAs (lncRNAs) and small ncRNAs.

LncRNAs are transcripts longer than 200 nucleotides that interact with different biological macromolecules (DNA, chromatin and proteins) and RNAs (mRNAs, miRNAs and other lncRNAs) and play significant roles in various biological functions. LncRNAs can cis- or trans-regulate genes at both transcriptional, post-transcriptional and epigenetic levels. LncRNAs exert regulatory functions by interacting with chromatin remodeling complexes, RNA binding proteins and transcription factors. LncRNAs act through different mechanisms: guides to recruit epigenetic regulators on chromatin, decoy targets of bioactive molecules, guidance for the specific subcellular localization of proteins, scaffolds for intermolecular interactions (9). Numerous lncRNAs are involved in the complex regulatory network leading to CRC development, playing important role in the diagnosis, treatment and prediction of prognosis, as well as, in metastases and drug resistance (10).

Among the small ncRNA species, microRNAs (miRNAs) are by far the most extensively studied in cancer, and alterations of miRNAs have been demonstrated to play a critical role in affecting molecular and cellular features of cancer-associated malignant phenotypes.

MiRNAs act as regulators of gene expression at post-transcriptional level by inhibiting mRNA translation and/or by cleavage of their RNA targets. Growing evidence suggest that miRNAs play a pivotal role in the regulation of genes driving CRC initiation, progression and metastatic process. Some of them, including miR-21, miR-34 family, miR-155, miR-224, and miR-378, have been extensively studied in CRC having important roles in diagnosis, prognosis, and therapy (11).

Noteworthy, plasma level of miR-378 has the highest predictive capability in CRC and could be useful for discriminating CRC patients from healthy individuals (12). Moreover, a strong association between low levels of miR-378 and increased tumor volume, metastasis and short overall survival of CRC patients has been observed suggesting that miR-378 plays a critical role in carcinogenesis and could be used as a biomarker to predict the outcome of CRC patients (13).

Recent studies have demonstrated that members of miR-378 family, composed of eight miRNAs (miR-378i, miR-378a-3p, miR378c, miR-378d, miR-378e, miR-378f, miR-378a-5p, and miR-378g), are frequently downregulated in CRC (14, 15). Of note, miR-378a-3p, has been reported to have a crucial role in carcinogenesis acting as a tumor suppressor, promoting apoptosis and cell cycle arrest and reducing invasion and drug resistance in several human cancers such as prostate (16), liver (17), ovarian (18), breast cancer (19), glioblastoma (20) and esophageal squamous cell carcinoma (21).

MiR-378a-3p expression is significantly associated with histological differentiation grade, TNM stage, T classification and the prognosis of CRC patients, however little is known about its biological role and the underlying molecular mechanism in CRC tumorigenesis (15). In this study, we showed the downregulation of miR-378a-3p in CRC and in our collection of patient-derived CRC-SCs (22) supporting its role as tumor suppressor. By exploring the molecular mechanisms and the regulatory network underlying its function, we found two putative targets of miR-378a-3p: Metastasis Associated Lung Adenocarcinoma Transcript 1 (MALAT1) and Nuclear Enriched Abundant Transcript 1 (NEAT1), two lncRNAs known to have a critical role in CRC tumorigenesis. Our study provides a new potential therapeutic target, acting on both tumor bulk cells and the subpopulation of cancer stem-like cells, in the context of CRC carcinogenesis.

## Material and Methods

### CRC and Normal Mucosal Tissue Sample Collection and Cell Cultures

All human CRC tissues and normal mucosal samples were obtained from adult patients who underwent colorectal tumor resection at the Sant’Andrea Hospital in Rome. Written informed consent, in accordance with the research proposals approved by the Institutional Ethical Committee, was provided by all patients before surgery.

CRC-SC lines were isolated from surgical samples through mechanical and enzymatic dissociation using type II collagenase (Gibco Invitrogen by ThermoFisher Scientific, Waltham, MA, USA). The resulting single cell suspension was cultivated in a serum-free medium supplemented with 20 ng/ml epidermal growth factor (EGF) and 10 ng/ml basic fibroblast growth factor (bFGF) (PeproTech, London, UK). Stem cell marker CD133 and epithelial marker Ber-Ep4 expression was assessed by flow cytometry using the following antibodies: anti-Epithelial Antigen-FITC (clone Ber-Ep4, mouse IgG1, DakoCytomation, Denmark), anti-CD133-PE (clone AC133/1, mouse IgG1, MiltenyiBiotec Inc., Bergisch Gladbach, Germany), or isotype-matched control antibodies. CRC-SC lines were analyzed with FACSCanto flow cytometer (BD Biosciences, Milan, Italy) and data were analyzed with FACS Diva software (BD Biosciences), as previously described (6). To validate CRC-SC lines, Short Tandem Repeat (STR) DNA fingerprinting was performed. Nine highly polymorphic STR loci plus amelogenin (Cell ID™ System, Promega Inc., Madison, WI, USA) were used. Detection of amplified fragments was obtained by ABI PRISM 3100 Genetic Analyzer (Applied Biosystems by ThermoFisher Scientific). Data were analysed by GeneMapper^®^ software, version 4.0 (Biological Bank and Cell Factory, National Institute for Cancer Research, IST, Genoa, Italy). All CRC-SC line profiles were challenged against public databases to confirm authenticity (22).

Human CRC cell lines were purchased from American Type Culture Collection (ATCC) and cultivated in the recommended media (see www.atcc.org for details).

The packaging human embryonic kidney cell line, 293T, was purchased from the ATCC and maintained in DMEM (Euroclone, Pero, MI, Italy) supplemented with 10% heat inactivated FBS (Euroclone), 2 mM L-glutamine, 100 U/mL of penicillin and 100 µg/mL of streptomycin (Euroclone).

### RNA Extraction and Real-Time RT-PCR

Total RNA was isolated from cells using TRIzol reagent (Invitrogen by ThermoFisher Scientific). Real-time PCR for miR-378a-3p was carried out using TaqMan^®^ MicroRNA Assays protocol (assay ID 002243, Applied Biosystems) and normalized with RNU6B (assay ID 001093, Applied Biosystems). All RT-PCR reactions were run in duplicate in StepOnePlus™. Real-time PCR for EMT markers was carried out using PrimePCR™ precasted 96-well EMT pathway plate (Bio-Rad Laboratories, Inc. Hercules, CA, USA). Real-time PCR for MALAT1 and NEAT1 mRNA detection were performed with SYBR™ Green Master Mix in StepOnePlus™ Real-Time PCR System (Applied Biosystems) and normalized with GAPDH using the following primers:

**Table:** 

	Oligo For: 5’->3’	Oligo Rev: 5’->3’
**MALAT1**	GTGTGCCAATGTTTCGTTTG	AGGAGAAAGTGCCATGGTTG
**NEAT1**	TGCTTGTTCCAGAGCCCATGAATGCCA	GTTCTACAGCTTAGGGATCTTCTTGAAGC
**GAPDH**	ACCTGACCTGCCGTCTAGAAAA	CCTGCTTCACCACCTTCTTGA

### Plasmid Constructs and Lentivirus Infection

The miR-378a-3p precursor, from HCT116, was cloned in the 3’-untranslated (UTR) region of RFP in a doxycycline-inducible Tet-On lentiviral vector (TRIPZ) (ThermoFisher Scientific). Primers used for pri-miRNA-378a-3p amplification were: 5’-TTAACGCGTCATGACAGGCCGAGGATCTTCTGGTGAT-3’ (Forward) and 5’-AAACTCGAGCCCCAATGGGTAAAAGTTAAGTCTCCT-3’ (Reverse).

For *in vivo* imaging, the luciferase cDNA was cloned into pRRLsin.cPPT.hCMV.hPGK.GFP (LUC-GFP) vector by XbaI-Xho restriction enzyme (23).

Lentiviral particles were produced in the packaging cell line 293T (at 70-80% confluency) using the calcium phosphate transfection protocol and infection was performed as previously described (23). After infection, TRIPZ or TRIPZ-miR-378 transduced cells were selected with puromycin (Sigma-Aldrich Inc., Saint Louis, MO, USA) and RFP fluorescence was assessed by FACSCanto (BD Biosciences) upon doxycycline induction (Sigma-Aldrich Inc.). Then, RFP-positive cells were flow sorted by FACS Aria (BD Biosciences).

### Cell Growth, Migration, and Colony Formation Assay

For the viability assay, TRIPZ and TRIPZ-miR378 transduced CRC cell lines (i.e., HCT116, HT29 and DLD1) and CRC-SCs were plated at density of 6-8x10^3^/mL and 2x10^4^/mL, respectively, in 96-well plates in triplicate. Cell viability was evaluated using the CellTiter-Blue Viability Assay (Promega Inc.) following the manufacturer’s instruction. Briefly, 20 μl of the CellTiter-Blue^®^ Reagent was added directly to each well, the plates were incubated for 1h at 37°C to allow viable cells to reduce the indicator dye resazurin into the highly fluorescent resorufin and the fluorescent signal was measured by MultiLabel Plate Reader VICTOR X3™ (Perkin Elmer, Norwalk, USA).

For migration assay, we used the Corning FluoroBlok™ Multiwell Inserts System (Corning Life Sciences, Tewksbury, MA, USA), following the manufacturer’s instruction. In this plate, each well is divided in two chambers, the upper one and the lower one, separated by a polyethylene terephthalate (PET) insert. Briefly, 1.5x10^3^ transduced CRC cell lines and 3x10^3^ transduced CRC-SCs were plated in the upper chamber in recommended medium without heat inactivated FBS or growth factors (EGF and bFGF). Medium supplemented with 10% heat inactivated FBS or stem cell medium supplemented with growth factors (EGF and bFGF) was added in the lower chamber and used as chemoattractant to promote migration. After 48h, the fluorescent dye calcein acetoxymethyl ester (calcein-AM, Life Technologies Corporation by ThermoFisher Scientific) was added to the lower chamber and incubated at 37°C for 30 min. The cell viability indicator, calcein-AM, is a non-fluorescent, cell permeate compound that is hydrolyzed by intracellular esterases into the fluorescent anion calcein and can be used to fluorescently label viable cells before microscope observation. The number of migrated cells was evaluated by counting the cells after imaging acquisition using a fluorescence microscope.

Colony formation ability of transduced CRC cell lines was assessed by plating 1x10^3^ cells in 6-well plates. After 1 week, samples were fixed at room temperature using 2.5% glutaraldehyde for 20 min and stained with 0.1% crystal violet for 20 min. After rinsing three times, colonies were observed and analyzed using ImageJ version 1.8.0 software (National Institutes of Health).

Colony formation ability of transduced CRC-SCs was assessed by plating a single cell/well in 96-well plates. After 3-4 weeks, each well was analysed and the number of spheres/cell aggregates was counted. All the experiments were performed in medium in the presence of doxycycline.

### Reporter Assay

The human MALAT1 sequences containing the target site for miR-378a-3p (wild-type and mutated) were synthesized and cloned into pGL3 control vector (Promega Inc) downstream of the luciferase reporter gene.

**Table:** 

		3’ -> 5’
**MALAT1 wt**	For	AGCGAGCTCGCAGCAGTTCGTGGTGAAGATAGGAAAAGAGTCCAGGAGCCAGTGCGATTT
	Rev	AAATCGCACTGGCTCCTGGACTCTTTTCCTATCTTCACCACGAACTGCTGCGAGCTCGCT
**MALAT1 mt**	For	GAGCTCGCAGCAGTTCGTGGTGAAGATAGGAAAAGTCAGGTCGAGCCAGTGCGAT
	Rev	CTAGATCGCACTGGCTCGACCTGACTTTTCCTATCTTCACCACGAACTGCTGCGAGCTC

TRIPZ and TRIPZ-miR-378 DLD1 cells were transiently transfected by Lipofectamine 2000 (Invitrogen), with 400 ng of luciferase reporter plasmid containing wild-type or mutated MALAT1-seeds. 36h post-transfection luciferase activity was quantified by Dual Luciferase Reporter kit (Promega Inc.)

### RNA Immunoprecipitation

RIP assay was performed by using the miRNA Target IP Kit (Active Motif, Carlsbad, CA). Lysates from 10^6^ TRIPZ or TRIPZ-miR-378 DLD1 transduced cells were incubated with protein G magnetic beads and a pan-Ago antibody that recognizes Ago 1/2/3 according to manufacturer’s instruction. Then, precipitated RNAs were purified, and RT-PCR was performed to detect the levels of the two lncRNAs and Filamin B (FLNB) as a miR-378a-3p non-target mRNA, (FLNB For: ACACCAAAGCTGCAGGAAGT; FLNB Rev: GGCTCTTTGGAATGTGGTGT).

### CRC-SC Xenograft Mouse Models

Animal experiments were performed in accordance with relevant institutional and national regulations. For subcutaneous xenografts, 2.5x10^5^ CRC-SCs co-infected with LUC-GFP and TRIPZ (empty) or TRIPZ-miR-378 vectors were resuspended in matrigel^®^ (Corning Life Sciences) and injected in the flanks of NOD/SCID mice (n, 4; 4–6 weeks of age; CD1 NOD-/SCID mice, Charles Rives, Italy). LUC-GFP TRIPZ cells were injected in one flank whereas LUC-GFP TRIPZ-miR-378 cells were injected in the other flank. Doxycycline administration in drinking water (200 μg/ml) started from the day of injection. Tumor size was monitored by measuring the length (L) and width (W) with a caliper. Volumes were calculated by using the following formula: (L x W^2^) x 0.52 and statistical significance was calculated using Two-tailed Student’s t-test.

For liver xenografts, CRC-SCs co-infected with LUC-GFP and TRIPZ or TRIPZ-miR-378 vectors were resuspended in 50% matrigel^®^ (Corning Life Sciences)/50% medium; 2.5x10^5^ cells were injected into the liver of NOD/SCID mice (n, 5; 4–6 weeks of age; CD1 NOD-/SCID mice, Charles Rives).

During the experiments, doxycycline was administered to the mice in drinking water (200 μg/ml) starting from the day of injection.

For *in vivo* imaging analysis, mice were injected intraperitoneally with 150 mg/kg D-luciferin (Caliper Life Sciences, Waltham, MA, USA) 10 min before imaging and then were sedated with 20 mg/kg valium. Data acquisition was performed by IVIS 100 Imaging System (Perkin Elmer). Whole animal imaging was used to monitor tumor growth; signal intensities were quantified as the sum of all detected photons.

### Automated Capillary Western Immunoassay

Cellular proteins were extracted by using Nonidet-P40 lysate buffer (1% NP40, 200 nM NaCl, 50 mM Tris pH 7.4) with addition of protease and phosphatase inhibitors (Sigma-Aldrich Inc.), maintained on ice for 30 min, vortexed and then centrifuged at 12000 rpm for 10 min at 4°C. Protein concentration was determined using BioRad protein-assay (Bio-Rad Laboratories, Inc.). Analysis of protein expression was performed using capillary WES™ technology by ProteinSimple (Bio-Techne, Minneapolis, MN, USA) according to the manufacturer’s protocol. 500 ng of each sample were added to the fluorescent 5XMaster Mix and loaded into a Wes 12–230 kDa plate, together with a biotinylated ladder, primary antibodies, ready-to-use HRP-conjugated anti-rabbit or anti-mouse secondary antibody and luminol-peroxide mix. The acquisition and quantitative analysis of images were executed with Compass software (ProteinSimple). The following primary antibodies were used: 1:25 anti-Jagged-1 (#2620, Cell Signaling, Danvers, MA, USA), 1:30 anti-pAKT(Ser473)(#4058, Cell Signaling), 1:75 anti-AKT (#9272, Cell Signaling) and 1:100 anti-β-actin (A5441, Sigma-Aldrich Inc.).

### Statistical Analysis

GraphPad prism v4.0 (GraphPad Software, La Jolla, CA, USA, www.graphpad.com) was used for statistical analysis. Two-tailed Student’s t-test was used to evaluate statistical significance, the level of significance is indicated in the plots using asterisks as follows: * for p<0.05, ** for p<0.01 and *** for p<0.001.

## Results

### MiR-378a-3p is Downregulated in CRC Tissues and Patient-Derived CRC-SC Lines

**Figure 1:**
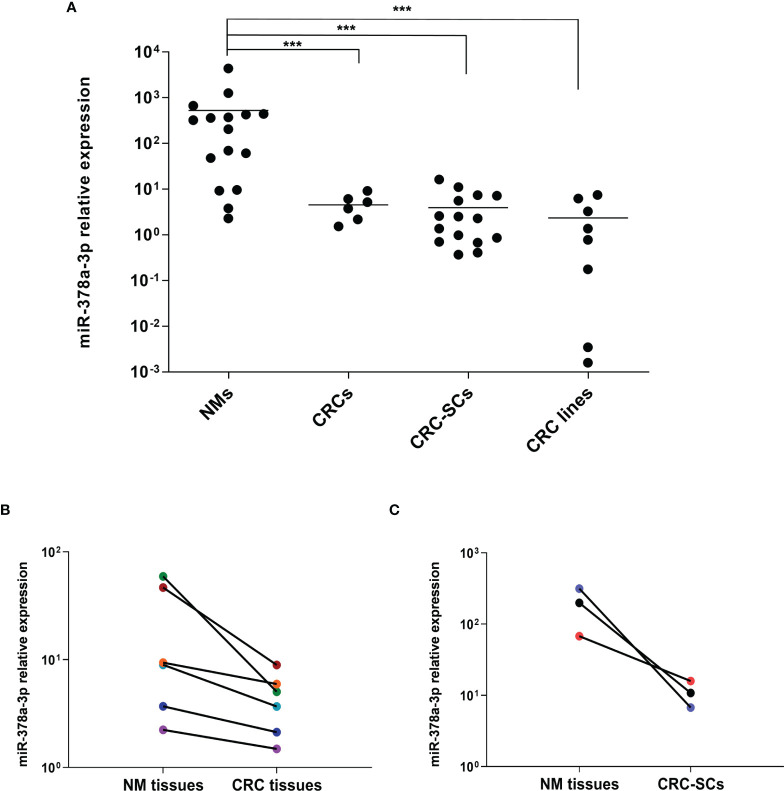
miR-378a-3p is low expressed in CRC tissues, patient-derived CRC SCs and CRC cell lines. **(A)** MiR-378a-3p expression was analyzed by qRT-PCR in normal mucosal tissues (NMs, n=16), CRC tissues (CRCs, n=6), CRC-SCs (n=15) and CRC cell lines (n=8). Samples were run in duplicate. Data were normalized to the small nuclear RNA RNU6B expression in the corresponding samples. ***, p<0.001 based on Mann-Whitney test. **(B)** qRT-PCR analysis of miR-378a-3p expression in 6 matched pairs of CRC tissues and normal mucosal tissues derived from the same patient (same color points). **(C)** qRT-PCR analysis of miR-378a-3p expression in 3 matched pairs of CRC-SC lines and normal mucosal tissues derived from the same patient (same color points). Samples were run in duplicate. Data were normalized to the small nuclear RNA RNU6B expression in the corresponding samples.

MiR-378a-3p expression was often found downregulated in CRC, and its low expression was associated with clinico-pathological features and poor prognosis of CRC patients (14, 15). To evaluate whether miR-378a-3p was downregulated in our cohort of patients, we analyzed its expression in a panel of 6 CRC tissues (CRCs), 15 colorectal cancer stem-like cell (CRC-SC) lines isolated from tumor tissues of CRC patients (22), 8 CRC cell lines and 16 normal mucosal tissues (NMs) by qRT-PCR. As shown in **Figure 1A**, miR-378a-3p expression is significantly lower in CRC tissues (p=0.0012), CRC-SCs (p<0.001) and CRC cell lines (p<0.001), than in normal mucosal tissues. Noteworthy, each CRC tissue expresses less miR-378a-3p than the adjacent normal mucosal tissue (**Figure 1B**), as well as, the 3 CRC-SC lines for which a normal mucosa from the same patient was available (**Figure 1C**). These results confirmed that miR-378a-3p is downregulated in CRC, suggesting that its aberrant expression may contribute to CRC pathogenesis.

### MiR-378a-3p Restoration Restrains Tumorigenic Properties of CRC Cell Lines and Patient-Derived CRC-SC Lines

**Figure 2:**
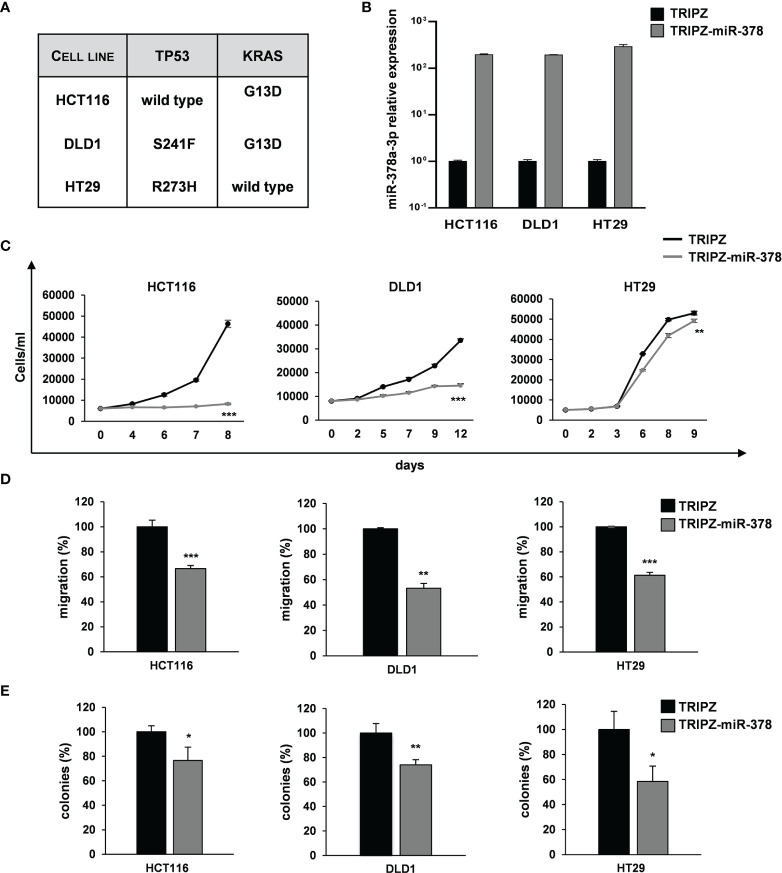
miR-378a-3p restoration reduces cell growth, migration and clonogenic abilities of CRC cell lines. **(A)** Mutation status of CRC critical genes in selected CRC cell lines (HCT116, DLD1 and HT29). **(B)** MiR-378a-3p expression was analyzed by qRT-PCR in HCT116, DLD1 and HT29 transduced with TRIPZ or TRIPZ-miR-378 inducible vectors after doxycycline exposure. **(C)** Growth curves of HCT116, DLD1 and HT29 cells transduced with either TRIPZ or TRIPZ-miR-378 vector. Points and range lines at each day represent mean ± SD of at least two independent experiments in triplicate. **(D)** Migration assay of HCT116, DLD1 and HT29 cells transduced with TRIPZ or TRIPZ-miR-378 vector. Values are reported as percentage relative to control vector and shown as mean ± SD from two independent experiments in triplicate. **(E)** Colony formation assay of HCT116, DLD1 and HT29 cells transduced with TRIPZ or TRIPZ-miR-378 vector. Percent colony number values from two independent experiments in triplicate were calculated over the correspondent TRIPZ vector and are shown as mean ± SD for each CRC cell line. *p<0.05, **p<0.01; ***p<0.001 based on Student’s t test.

To explore the role of miR-378a-3p in CRC tumorigenesis, we restored its expression in CRC cell lines using a doxycycline-inducible Tet-On lentiviral vector (TRIPZ) carrying pri-miR-378a in the 3’ untranslated (UTR) region of Red Fluorescent Protein (RFP) gene (TRIPZ-miR-378). We transduced three CRC cell lines (HCT116, DLD1 and HT29) selected as representative of different mutation status of CRC critical genes (i.e., TP53 and KRAS). Particularly, HCT116 with TP53 wild type and KRAS mutated, DLD1 with both TP53 and KRAS mutated and HT29 with TP53 mutated and KRAS wild type (24) (**Figure 2A**).

After doxycycline induction, RFP-positive cells were flow sorted and the miR-378a-3p restoration was confirmed by qRT-PCR analysis (**Figure 2B**). Its expression was increased in all the TRIPZ-miR-378 CRC cell lines compared to cells transduced with TRIPZ empty vector. Thus, we analyzed the effect of miR-378a-3p restoration on tumorigenic properties of CRC cell lines.

Ectopic expression of miR-378a-3p significantly reduced the growth of TRIPZ-miR-378 cells compared to TRIPZ cells in all the CRC cell lines tested (**Figure 2C**). Then, we investigated the effect of miR-378a-3p on the migration and colony formation ability of CRC cell lines. As shown in **Figures 2D, E**, we observed a considerable reduction in the motility and colony formation ability of TRIPZ-miR-378 CRC cell lines compared to TRIPZ cells. Altogether these results showed that miR-378a-3p restoration impaired tumorigenic properties of all the CRC cell lines analyzed, suggesting that this miRNA may function as tumor suppressor in CRC tumorigenesis.

**Figure 3:**
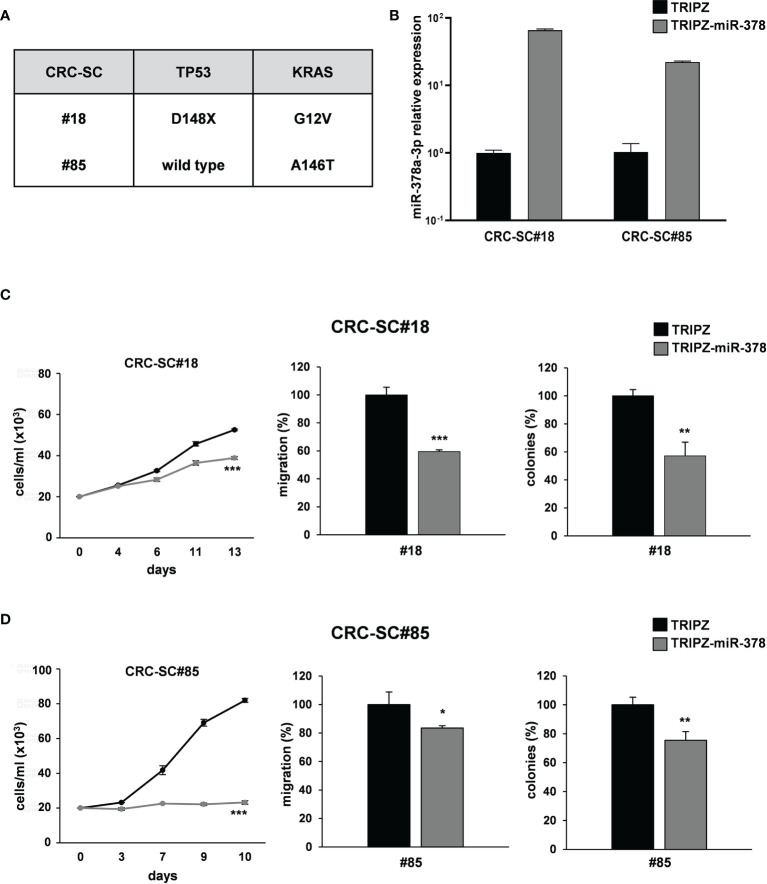
miR-378a-3p restoration reduces cell growth, migration and clonogenic abilities of patient-derived CRC-SCs. **(A)** Mutation status of CRC critical genes in patient-derived CRC-SCs (#18 and #85). **(B)** MiR-378a-3p expression was analyzed by qRT-PCR in CRC-SC#18 and CRC-SC#85 transduced with TRIPZ or TRIPZ-miR-378 inducible vectors after doxycycline exposure. Functional *in vitro* assays on **(C)** CRC-SC#18 and **(D)** CRC-SC#85 transduced with TRIPZ or TRIPZ-miR-378 vector. Growth curves of CRC-SCs (*left panels*). Points and range lines at each day represent mean ± SD of at least two independent experiments in triplicate. Migration assay of CRC-SCs transduced with TRIPZ or TRIPZ-miR-378 vector (*center panels*). Values are reported as percentage relative to TRIPZ vector and shown as mean ± SD from two independent experiments in triplicate. Colony formation assay of CRC-SCs transduced with TRIPZ or TRIPZ-miR-378 vector (*right panels*). Percent colony number values from two independent experiments in triplicate were calculated over the correspondent TRIPZ vector and are shown as mean ± SD for each CRC-SC line. *p<0.05; **p<0.01; ***p<0.001 based on Student’s t test.

To corroborate these results, we analyzed whether miR-378a-3p could also affect tumorigenic ability of our collection of patient-derived CRC-SC lines. Two CRC-SC lines, CRC-SC#18 (TP53 and KRAS mutated) and CRC-SC#85 (TP53 wild type and KRAS mutated) (**Figure 3A**), were transduced using the lentiviral vectors TRIPZ or TRIPZ-miR-378 (25). After doxycycline induction, RFP-positive CRC-SCs were flow sorted and miR-378a-3p expression was confirmed by qRT-PCR analysis (**Figure 3B**). As expected, miR-378a-3p restoration led to reduced proliferation and migration ability of both CRC-SC lines transduced with TRIPZ-miR-378 compared to TRIPZ transduced cells (**Figures 3C, D**). In addition, we evaluated whether miR-378a-3p could alter the clonogenic ability of CRC-SC lines. We observed that miR-378a-3p restoration inhibited the clonogenic ability of CRC-SCs, as demonstrated by fewer colonies in CRC-SCs transduced with TRIPZ-miR-378 compared to control cells (**Figures 3C, D**). Taken together, our results strengthened the hypothesis that miR-378a-3p is involved, as tumor suppressor, in CRC tumorigenesis.

Since functional *in vitro* assays showed that miR-378a-3p restoration induced a significantly reduction of migration abilities in both CRC cell and CRC-SC lines, we analyzed the expression of a panel of mesenchymal and epithelial marker genes on TRIPZ and TRIPZ-miR-378 transduced CRC cell lines (HT29, DLD1 and HCT116) and CRC-SC lines (CRC-SC#18 and CRC-SC#85) by qRT-PCR analysis. The mRNA levels of several mesenchymal marker genes (TGFB1, TGFB2, SNAI1 and beta-catenin) were reduced in both TRIPZ-miR-378 CRC cell lines and CRC-SCs compared to TRIPZ cells (**Supplementary Figure S1**).

Among these genes (**Supplementary Figure S1**), Jagged-1 (JAG-1), involved in Notch signaling pathway, and AKT1, a critical player in tumorigenesis, were both predicted as direct targets of miR-378a-3p even if with low stringency, by *in silico* analysis (targetscan.org; starbase.sysu.edu.cn).

To verify whether AKT1 and JAG1 were direct targets of miR-378a-3p, we performed dual-luciferase reporter assay using TRIPZ-miR-378 or TRIPZ vector transduced DLD1 cells and reporter vectors containing the wild type or mutated seed of these genes. However, we observed no difference in the luciferase activity of the wild type reporter vector compared to the mutated reporter construct (data not shown). Although in DLD1 cell context, AKT and JAG1 are not direct targets of miR-378a-3p we can not exclude an indirect regulation or a direct interaction in other cell contexts.

### MiR-378a-3p Interacts With MALAT1 and NEAT1 and Their Expression is Inversely Correlated in CRC-SC Lines

To evaluate how miR-378a-3p could be involved in the regulation of these genes we analysed possible interaction between miR-378a-3p and lncRNAs as critical regulators of gene expression and epigenetic mechanisms, affecting several cancer-related events.

**Figure 4:**
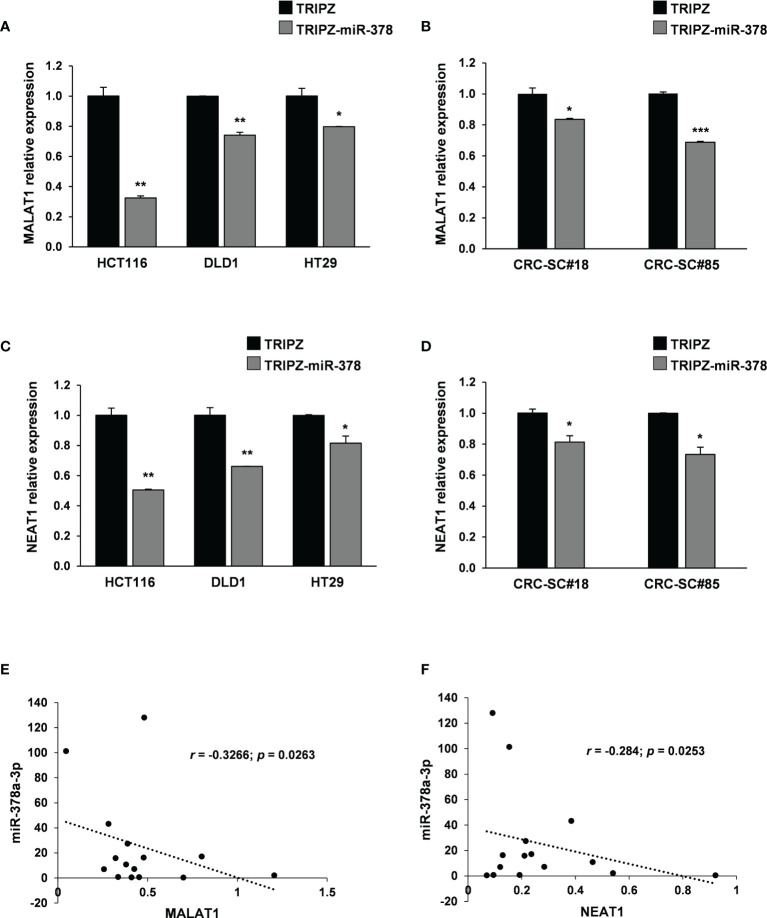
MALAT1 and NEAT1 are modulated by miR-378a-3p and their expression is inversely correlated in CRC-SCs. MALAT1 expression was analyzed by qRT-PCR in **(A)** CRC cell lines (HCT116, DLD1 and HT29) and **(B)** CRC-SC lines (CRC-SC#18 and CRC-SC#85) transduced with TRIPZ or TRIPZ-miR-378 inducible vectors after doxycycline exposure. qRT-PCR analysis for MALAT1 confirmed the downregulation of the lncRNA. NEAT1 expression was analyzed by qRT-PCR in **(C)** CRC cell lines (HCT116, DLD1 and HT29) and **(D)** CRC-SC lines (CRC-SC#18 and CRC-SC#85) transduced with TRIPZ or TRIPZ-miR-378 inducible vectors after doxycycline exposure. qRT-PCR analysis for NEAT1 confirmed the downregulation of the lncRNA. **(E)** Correlation analysis between miR-378a-3p and MALAT1 expression levels in CRC-SC lines (p=0.0263). **(F)** Correlation analysis between miR-378a-3p and NEAT1 expression levels in CRC-SC lines (p= 0.0253).

To this end, we analyzed possible interaction between miR-378a-3p and lncRNAs as they regulate several cancer-related events. We performed *in silico* analysis using dedicated algorithms (starBase v2.0), and among the best predicted ncRNA target of miR-378a-3p, we identified MALAT1. It has been reported that the lncRNA MALAT1, promotes proliferation, metastasis, and epithelial-to-mesenchymal transition (EMT) through multiple signaling pathways in several cancers, including CRC (26–28). As first validation of this potential interaction, we observed by qRT-PCR analysis that ectopic expression of miR-378a-3p significantly downmodulated MALAT1 expression in both CRC cells (HT29, DLD1 and HCT116) and CRC-SC lines (CRC-SC#18 and CRC-SC#85) transduced with TRIPZ-miR-378 compared to TRIPZ transduced cells (**Figures 4A, B**). To verify the potential direct interaction between these ncRNAs, we performed dual-luciferase reporter assay using TRIPZ-miR-378 or TRIPZ vector transduced DLD1 cells and reporter vectors containing the wild type or mutated seed of MALAT1. The reduction of the luciferase activity of wild type MALAT1 reporter vector but not of the mutated MALAT1 reporter construct confirmed the direct interaction between these ncRNAs (p<0.001; n=3) (**Supplementary Figure S2A**).

Growing evidences have identified NEAT1 lncRNA as an independent risk factor for poor prognosis of CRC. Particularly, high NEAT1 expression has been observed in CRC which contributes to the migration and invasion of CRC cells by sponging miR-185-5p, thus, upregulating IGF2 (29). Moreover, it has been shown that NEAT1 significantly promotes cell proliferation and migration of cardiomyocytes by interacting with miR-378a-3p (30). To verify this interaction in our cellular model, we analyzed NEAT1 expression by qRT-PCR in CRC cell lines (HCT116, DLD1 and HT29) and CRC-SC lines (CRC-SC#18 and CRC-SC#85) transduced with TRIPZ or TRIPZ-miR-378 inducible vectors after exposure to doxycycline (**Figures 4C, D**). As expected, miR-378a-3p restoration induced a significant downregulation of NEAT1 suggesting that the two ncRNAs may interact directly. Moreover, an inverse correlation between miR-378a-3p with both MALAT1 and NEAT1 expression was observed in our collection of patient-derived CRC-SC lines (p=0.0263 and p=0.0253, respectively) (**Figures 4E, F**).

To evaluate the putative direct binding between miR-378a-3p and NEAT1 or MALAT1, we performed RNA immunoprecipitation assays (RIP) using a pan-Ago antibody to pulldown the endogenous NEAT1 and MALAT1 associated to miRNA by using TRIPZ- and TRIPZ-miR-378 transduced DLD1 cell line (**Supplementary Figure S2B**). qRT-PCR on RNA recovered after RIP assay showed a fold-enrichment for both MALAT1 and NEAT1 between the TRIPZ- and the TRIPZ-miR-378-transduced DLD1 cells, not evident enough to definitively support a direct interaction between miR-378a-3p and the two lncRNAs.

Analyzing the location of miR-378a-3p and NEAT1 or MALAT1 by qRT-PCR in nuclear and cytoplasmic compartments of DLD1 cell line (**Supplementary Figure S3A**), we observed that the three ncRNAs mainly co-localized in the nucleus.

Furthermore, we analysed the expression of miR-378a-3p, NEAT1 and MALAT1in six CRC tissues and in six normal mucosal tissues. These results showed that a lower expression of miR-378a-3p is associated with a higher expression of the two lncRNAs in CRC tissues compared to NM tissues (**Supplementary Figure S3B**).

### MiR-378a-3p Restoration Significantly Reduces Tumor Growth in CRC-SC Xenograft Mouse Models

**Figure 5:**
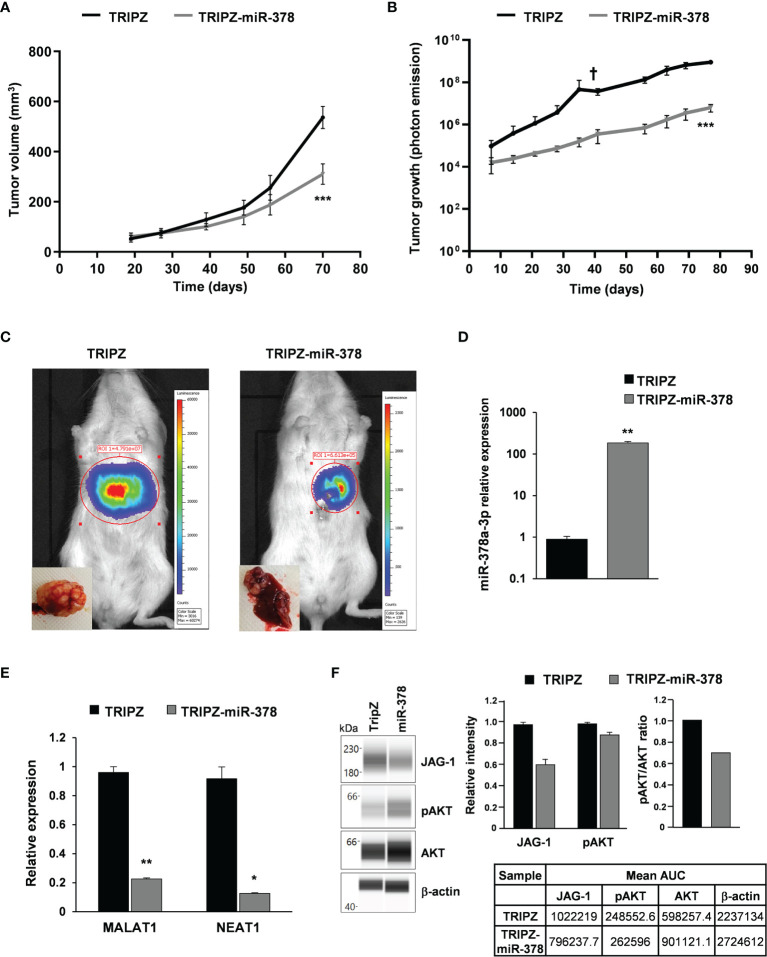
miR-378a-3p restoration reduces tumor growth of patient-derived CRC-SC xenografts. **(A)** Tumor growth curves of subcutaneous xenografts generated by the injection of CRC-SC#18 LUC-GFP TRIPZ (n=4) or LUC-GFP TRIPZ-miR-378 (n=4). Values represent volume (mm^3^) of xenograft tumors at different time points after injection. Values are reported as mean ± SD. Statistical comparison is reported for the latest time point and was calculated by means of Student’s t-test. ***p<0.001. **(B)** Tumor growth curves of liver xenografts generated by the injection of CRC-SC#18 LUC-GFP TRIPZ (n=5) or LUC-GFP TRIPZ-miR-378 (n=5). Values represent luciferase photon emission of xenograft tumors at different time points after injection. Values are reported as mean ± SD. Statistical comparison is reported for the latest time point and was calculated by means of Student’s t-test. ***p<0.001. † indicates the death of one mouse xenografted with CRC-SC#18 LUC-GFP TRIPZ 41 days after injection. **(C)** Representative images showing the bioluminescent signal of the CRC-SC#18 LUC-GFP TRIPZ (*left*) and CRC-SC#18 LUC-GFP TRIPZ-miR-378 (*right*) liver tumor xenografts acquired by IVIS imaging system at 41 days after injection. Representative images of the liver excised at the end of the experiment (*bottom left*). **(D)** MiR-378a-3p expression was analyzed by qRT-PCR in CRC-SC#18 LUC-GFP TRIPZ or LUC-GFP TRIPZ-miR-378 retrieved by fluorescent-activated cell sorting from liver tumor xenografts. **(E)** MALAT1 and NEAT1 expression was analyzed by qRT-PCR in CRC-SC#18 LUC-GFP TRIPZ or LUC-GFP TRIPZ-miR-378 retrieved by fluorescent-activated cell sorting from liver tumor xenografts. *p<0.05, **p<0.01, based on Student’s t test. **(F)** Wes full-length gel image of JAG-1, pAKT and AKT expression in CRC-SC#18 LUC-GFP TRIPZ or LUC-GFP TRIPZ-miR-378 retrieved by fluorescent-activated cell sorting from liver tumor xenografts (*left panel*). The relative amount of each immunoreactive band and the pAKT/AKT ratio was shown (*right panel*). The protein expression was quantified using AUC (area under the curve) measurements generated using Compass Software. Signal intensity was normalized to the β-actin level in the same sample.

Compared to differentiated cell lines, CRC-SCs represent improved preclinical models because of their unique capability to mimic tumor development *in vivo* as assayed by the ability of these cells to generate tumors that reproduce the phenotypic and histologic features of parental human counterparts (6). Therefore, we evaluated the effect of miR-378a-3p restoration in tumor xenografts generated by the injection of CRC-SC#18 transduced with empty vector (LUC-GFP TRIPZ) or miR-378a-3p vector (LUC-GFP TRIPZ-miR-378) into immunodeficient mice. Expression of transgene was induced during the experiments, by administration of doxycycline in drinking water starting from the day of injection. Subcutaneous xenografts generated by the injection of LUC-GFP TRIPZ-miR-378 cells displayed a significant reduction of the tumor growth rate (p<0.001, Student’s t-test) compared to those generated by empty vector transduced cells (**Figure 5A**).

To evaluate whether restoration of miR-378 was able to impair tumor growth also in an alternative model of CRC, we generated liver xenografts by intrahepatic injection of CRC-SC#18 LUC-GFP TRIPZ or LUC-GFP TRIPZ-miR-378 transduced cells (**Figure 5B**). Tumor growth curves, realized by evaluation of luciferase photon emission of xenograft tumors at different time points after injection, showed a significant growth reduction into the liver of LUC-GFP TRIPZ-miR-378 cells compared to LUC-GFP TRIPZ cells (p<0.001, Student’s t-test) (**Figures 5B, C**).

Successful and stable restoration of the miR-378a-3p expression was assessed by qRT-PCR in CRC-SC#18 LUC-GFP TRIPZ or LUC-GFP TRIPZ-miR-378 cells retrieved by fluorescent-activated cell sorting from liver tumor xenografts (**Figure 5D**).

To explore the molecular determinants of the effects of miR-378a-3p restoration *in vivo*, MALAT1 and NEAT1 expression was analyzed by qRT-PCR in CRC-SC#18 LUC-GFP TRIPZ or LUC-GFP TRIPZ-miR-378 retrieved by fluorescent-activated cell sorting from liver tumor xenografts. As shown in **Figure 5E**, MALAT1 and NEAT1 expression was significantly downregulated in CRC-SC#18 LUC-GFP TRIPZ-miR-378 cells retrieved from liver tumor xenografts compared to control cells (p<0.01 and p<0.05, respectively, Student’s t test). The stronger effect of miR-378 restoration observed in mice, in terms of MALAT1 and NEAT1 decreased expression, compared to that observed *in vitro*, could be due to a longer stimulation with doxycycline that may induce possible indirect regulation mechanisms that require longer time to be activated and observed.

Regarding the ncRNA downstream signaling pathways, we confirmed by Wes full-length gel image the reduced expression of JAG-1 and pAKT in CRC-SC#18 LUC-GFP TRIPZ-miR-378 compared to LUC-GFP TRIPZ cells retrieved by fluorescent-activated cell sorting from liver tumor xenografts (**Figure 5F**).

Taken together, these data confirmed the *in vitro* observations, reinforcing the hypothesis that the complex interplay between these three ncRNAs plays a pivotal role in the tumorigenic potential of CRC-SCs.

### MALAT1 and NEAT1 Knockdown Recapitulates the Effect of miR-378a-3p Restoration on CRC-SC Tumorigenicity

We investigated in our cellular model the individual contribution of the modulation of the two lncRNAs MALAT1 and NEAT1 in restraining the aggressive CRC-SCs’ behavior, *in vitro*.

**Figure 6:**
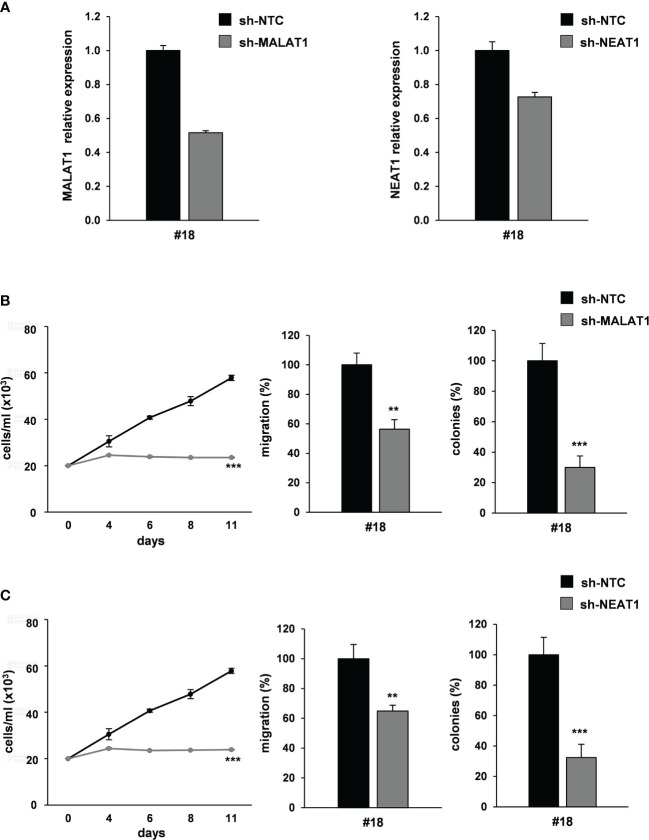
MALAT1 and NEAT1 knockdown recapitulates the effect of miR-378a-3p restoration on CRC-SC tumorigenicity. **(A)** qRT-PCR analysis for MALAT1 (*left panel*) and NEAT1 (*right panel*) on CRC-SC#18 transduced with sh-NTC, sh-MALAT1 or sh-NEAT1 vector confirmed lncRNA downregulation. **(B)** Functional *in vitro* assays: growth curve (*left panel*), migration assay (*center panel*) and colony formation assay (*right panel*) of CRC-SC#18 transduced with sh-NTC or sh-MALAT1 vectors. Values shown are mean ± SD from two independent experiments in triplicate. **p<0.01; ***p<0.001 based on Student’s t test. **(C)** Functional *in vitro* assays: growth curve (*left panel*), migration assay (*center panel*) and colony formation assay (*right panel*) of CRC-SC#18 transduced with sh-NTC or sh-NEAT1 vectors. Values shown are mean ± SD from two independent experiments in triplicate. **p<0.01; ***p<0.001 based on Student’s t test.

We silenced MALAT1 expression by transducing patient-derived CRC-SC#18 with a lentiviral vector carrying short hairpin (sh)-MALAT1 sequence and Green Fluorescent Protein (GFP) gene or a sh-NTC (no target control) lentiviral GFP vector. About a 40% decrease of the endogenous levels of MALAT1 was observed in GFP-positive cells after transduction (**Figure 6A**, *left panel*). Then, we analyzed the effect of MALAT1 silencing on CRC tumorigenic properties including proliferation, migration and clonogenic abilities *in vitro*. MALAT1 silencing induced a significant decrease of cell viability (**Figure 6B**, *left panel*) and of cell migration in CRC-SC#18 transduced with sh-MALAT1 compared to control cells (**Figure 6B**, *center panel*). Furthermore, a dramatic reduction of the clonogenic ability of CRC-SC#18 sh-MALAT1 compared to sh-NTC transduced cells (**Figure 6B**, *right panel*) was observed. Altogether, these results confirmed the role of this lncRNA as a critical determinant of CRC tumorigenesis.

Then, we analyzed the effect of NEAT1 knockdown by using a lentiviral vector carrying sh-NEAT1 or sh-NTC sequence and GFP as a reporter gene in CRC-SC#18. A 30% decrease of the endogenous levels of NEAT1 was observed in GFP-positive cells after transduction (**Figure 6A**, *right panel*). Furthermore, the effect of NEAT1 silencing on viability, clonogenic and migration abilities were evaluated, *in vitro* (**Figure 6C**). NEAT1 silencing was able to induce a significant decrease of cell viability (**Figure 6C**, *left panel*), migration (**Figure 6C**, *center panel*), and clonogenic abilities (**Figure 6C**, *right panel*) of stably NEAT1 silenced CRC-SCs compared to the control sh-NTC transduced cells, confirming the role of this lncRNA as a critical player of CRC tumorigenesis.

Taken together, our results highlight a complex interplay involving different species of RNAs, in which miR-378a-3p displays a tumor-suppressor function in CRC-SCs by modulating lncRNAs NEAT1 and MALAT1 that are critical for CRC cell growth and metastatization.

## Discussion

In this study we confirmed the involvement of miR-378a-3p in CRC and extended its role in promoting tumorigenic properties to CRC-SCs. Here we showed that restoration of miR-378a-3p expression inhibited cell growth, migration, and colony formation ability of patient-derived CRC-SCs, *in vitro* and decreased the growth of CRC-SC derived tumors, *in vivo*.

Our results also show that restoration of miR-378a-3p affects the expression of several mesenchymal and epithelial genes associated with EMT. The EMT process plays a significant and complex role in human CRC, where is closely associated with malignant behaviours, including tumor budding circulating cancer cell and drug resistance (31).

Particularly, we found that mRNA levels of several mesenchymal marker genes (TGFB1, TGFB2, SNAI1 and beta-catenin) were reduced in both CRC cell lines and CRC-SC lines after miR-378a-3p restoration. Particularly, JAG-1, involved in Notch signaling pathway, and AKT1, a critical player in tumorigenesis, were downmodulated in all the CRC cells and CRC-SC lines analyzed, even though, as luciferase reporter assay proved, neither JAG-1 nor AKT1 are miR-378a-3p direct targets, at least in the CRC cell line used for these analyses.

To evaluate how miR-378a-3p could be involved in the regulation of these genes we analysed possible interaction between this miRNA and lncRNAs.

Growing evidences have highlighted that miRNAs and lncRNAs do not act alone but interact with each other to regulate cellular processes for the maintenance of cellular homeostasis, consequently the disruption of these interactions can alter these processes contributing to cancer development.

Of note, miR-378 has already been described to be involved in complex regulatory networks with other ncRNA species (i.e. lncRNAs) in the tumorigenic context. It has been shown that miR-378 regulates proliferation and cell cycle progression of gastric cancer cells by targeting GAPLINC lncRNA whereas modulates apoptosis by interacting with GAS5 lncRNA in breast cancer (32, 33).

Our results showed that miR-378a-3p acts as a tumor suppressor in CRC-SCs affecting the expression of two lncRNAs MALAT1 and NEAT1.

Both MALAT1 and NEAT1, significantly overexpressed in many types of cancer have been identified as regulators in several biological process. They play roles in transcription and alternative splicing, and/or sponging miRNAs, which result in upregulation of genes involved in cancer-related events such as faster cell growth, angiogenesis, invasion, and migration (34, 35). Particularly, MALAT1 suppresses expression of anti-metastasis genes such as melanoma inhibitory activity 2 (MIA2), roundabout 1 (ROBO1), while induces pro-metastasis genes including latrophilin 2 (LPHN2) and ATP-binding cassette, subfamily A member (ABCA1) to promote metastasis (36). It has been reported that STAT3 may bind to the MALAT1 promoter and transcriptionally stimulate its expression in a model of prostate cancer identifying the IL-8/STAT3/MALAT1 axis as a key regulator during prostate tumorigenesis (37). MALAT1 has also been employed therapeutically in mouse models of lung and breast cancer where ASO-mediated silencing, significantly impairs tumor growth and metastasis formation (36, 38).

NEAT1 is frequently upregulated in cancer where exhibits an oncogenic role mainly by sponging tumor suppressive miRNAs, upregulating, in turn, oncogene expression. NEAT1 regulates gene expression and is crucial for the formation and maintenance of nuclear substructure paraspeckles. Interacting with several common genes and miRNAs in complex axis, NEAT1 regulates, other than normal cellular processes such as apoptosis and cell cycle, also tumorigenesis in glioblastoma (39). NEAT1 expression is closely related to the TNM stage, low survival rate and tumor recurrence in CRC thus, serving as an independent prognostic factor for tumor recurrence (40).

*NEAT1* and *MALAT1* loci are adjacent to one another on human chromosome 11q13.1 separated by less than 60 kb (41). Both MALAT1 and NEAT1 are abundant in the nucleus and localize at nuclear speckles and paraspeckles, respectively.

NEAT1 and MALAT1 co-localize to many genes, mainly over active genes, but show distinct binding patterns, at these sites, indicating different but synergistic functional roles for these RNAs on co-occupied genes (8).

According to a potential interaction between the two lncRNAs, MALAT1 knockout mice show variations in NEAT1 expression (42).

In the context of lung cancer, it has been recently demonstrated that OCT4, a key stemness transcription factor, promotes NEAT1 and MALAT1 transcription by targeting their promoter and enhancer regions (43). Furthermore, NEAT1 and MALAT1 function as downstream mediators of OCT4 to promote proliferation, migration and invasion of lung cancer cells (43).

Our data showed an example of the complex interplay between miR-378a-3p and the lncRNAs MALAT1 and NEAT1 and thus the disruption of this ncRNA regulatory network can alter the expression of genes involved in the EMT process (TGFB1, TGFB2, SNAI1, beta-catenin, AKT and JAG-1) in CRC.

Noteworthy, Akt signaling pathway was involved in regulating NEAT1 impact on induction of CRC cell growth, suggesting that NEAT1 enhanced AKT activation (44). More recently, it has been demonstrated that exosome-derived MALAT1 affects the malignant behavior of CRC cells by sponging miR-26a/26b *via* regulating FUT4 and activating PI3K/AKT/mTOR pathway (45).

The crosstalk between the lncRNAs, NEAT1 and MALAT1, and Notch pathway has been well documented in the context of oral squamous cell carcinoma where NEAT1 upregulates proliferation and EMT and represses apoptosis through activating VEGF-A and Notch signaling pathway (46) and in the context of chondrosarcoma for MALAT1 that contributes to the proliferation of tumor cells by activating Notch-1 signaling pathway (47).

Together, our data showed that miR-378a-3p may exert its tumor suppressor function modulating NEAT1 and MALAT1 lncRNA expression, though a weaker tumorigenic inhibition was observed in miR-378 restoration experiments compared to knockdown experiments. The stronger tumorigenic inhibition observed in sh-interference experiments, may be attributed either to a more efficient inhibitory effect of sh-constructs or to a fine-tuning role consistent with miRNA-regulation.

Anyway, our data identify miR-378a-3p as an important regulator of CRC tumorigenesis and suggest that it may be an interesting candidate as therapeutic agent, in the form of miRNA mimics, in colorectal cancer.

Given the recent success of RNAi-based and oligo-based drugs (48, 49), several therapeutic clinical trials with ncRNAs have begun (50) and, despite adverse effects of the first clinical experience with miR-34 replacement therapy (51), the correction of miRNA deregulation may be a promising therapeutic strategy for CRC treatment. Anyway it remains necessary to address toxicity concerns and resolve the bystander effects prior to large-scale clinical use.

## Data Availability Statement

The original contributions presented in the study are included in the article/**Supplementary Material**. Further inquiries can be directed to the corresponding author.

## Ethics Statement

The animal study was reviewed and approved by Italian Ministry of Health.

## Author Contributions

LR-V conceived and designed the study and wrote the manuscript. MBu and GC took care of CRC-SC cultures and carried out *in vitro* assays. VL and NF performed molecular data analysis. RI, FP, AB and GL contribute to the study by providing technical support. RI (molecular biology experiments), FP (WES experiments), AB (cell sorting and flow cytometry analysis) and GL (*in vivo* experiments). EP provided surgical specimens. MBi and GM supervised the study and contributed to the final version of the manuscript. All authors contributed to the article and approved the submitted version.

## Conflict of Interest

The authors declare that the research was conducted in the absence of any commercial or financial relationships that could be construed as a potential conflict of interest.

## Publisher’s Note

All claims expressed in this article are solely those of the authors and do not necessarily represent those of their affiliated organizations, or those of the publisher, the editors and the reviewers. Any product that may be evaluated in this article, or claim that may be made by its manufacturer, is not guaranteed or endorsed by the publisher.
