# Supplementary material for: MiR-378a-3p Acts as a Tumor Suppressor in Colorectal Cancer Stem-Like Cells and Affects the Expression of MALAT1 and NEAT1 lncRNAs
Source: Front Oncol. 2022 Jun 24;12:867886. doi: 10.3389/fonc.2022.867886 (PMC9263271; doi:10.3389/fonc.2022.867886)
Supplement: Supplementary file 1 [file DataSheet_1.pdf]

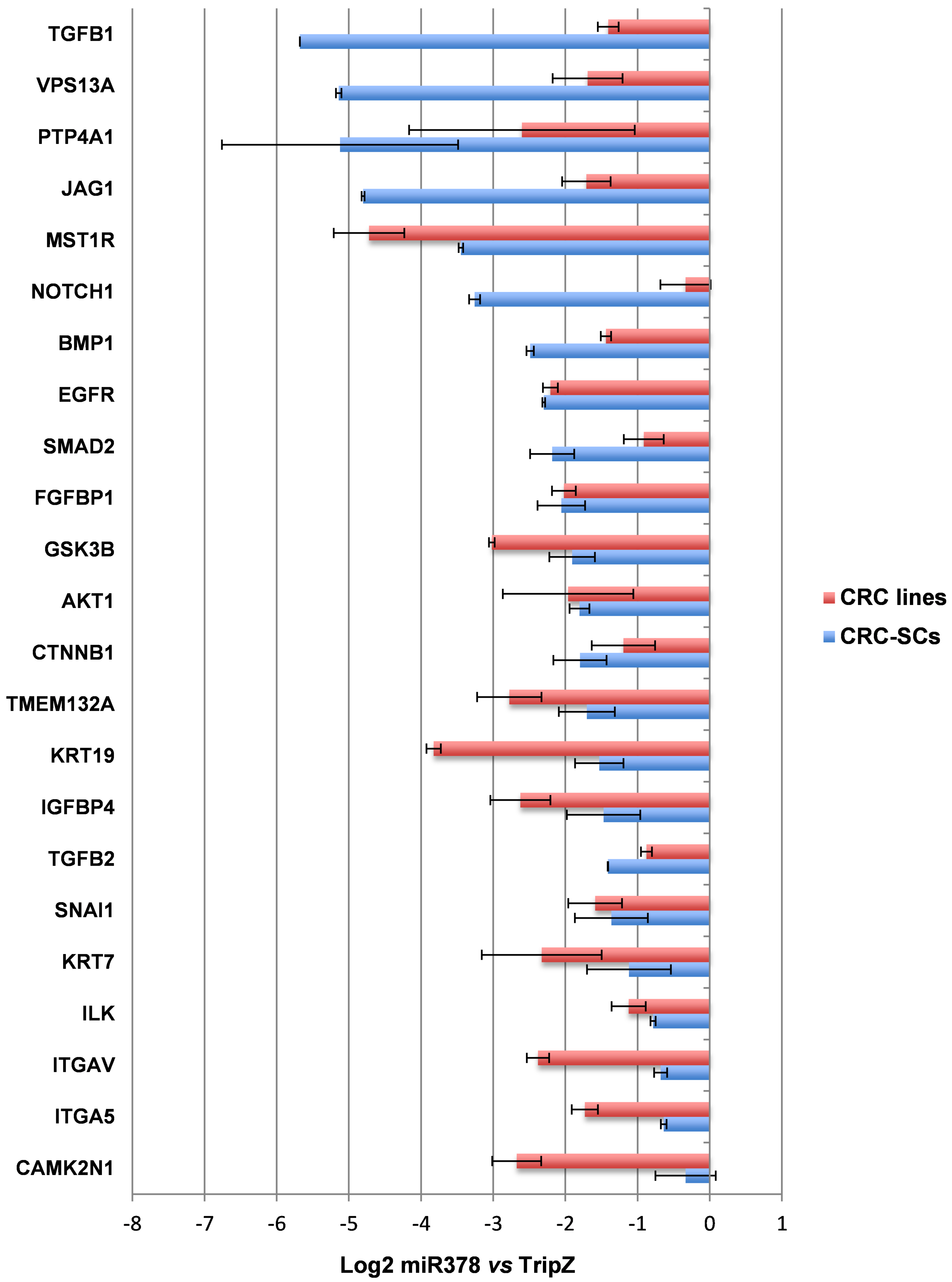

Supplementary Figure S1. RT-qPCR of modulated genes from PrimePCR^{TM} precasted EMT pathway plates. Data presented are Log_2 Fold Change media of relative normalized expression in overexpressing miR-378a cells vs TripZ control cells in CRC lines (HT116, HT29, DLD1) Red Bars and in CRC-SCs (#18, #85) Blue Bars.

A

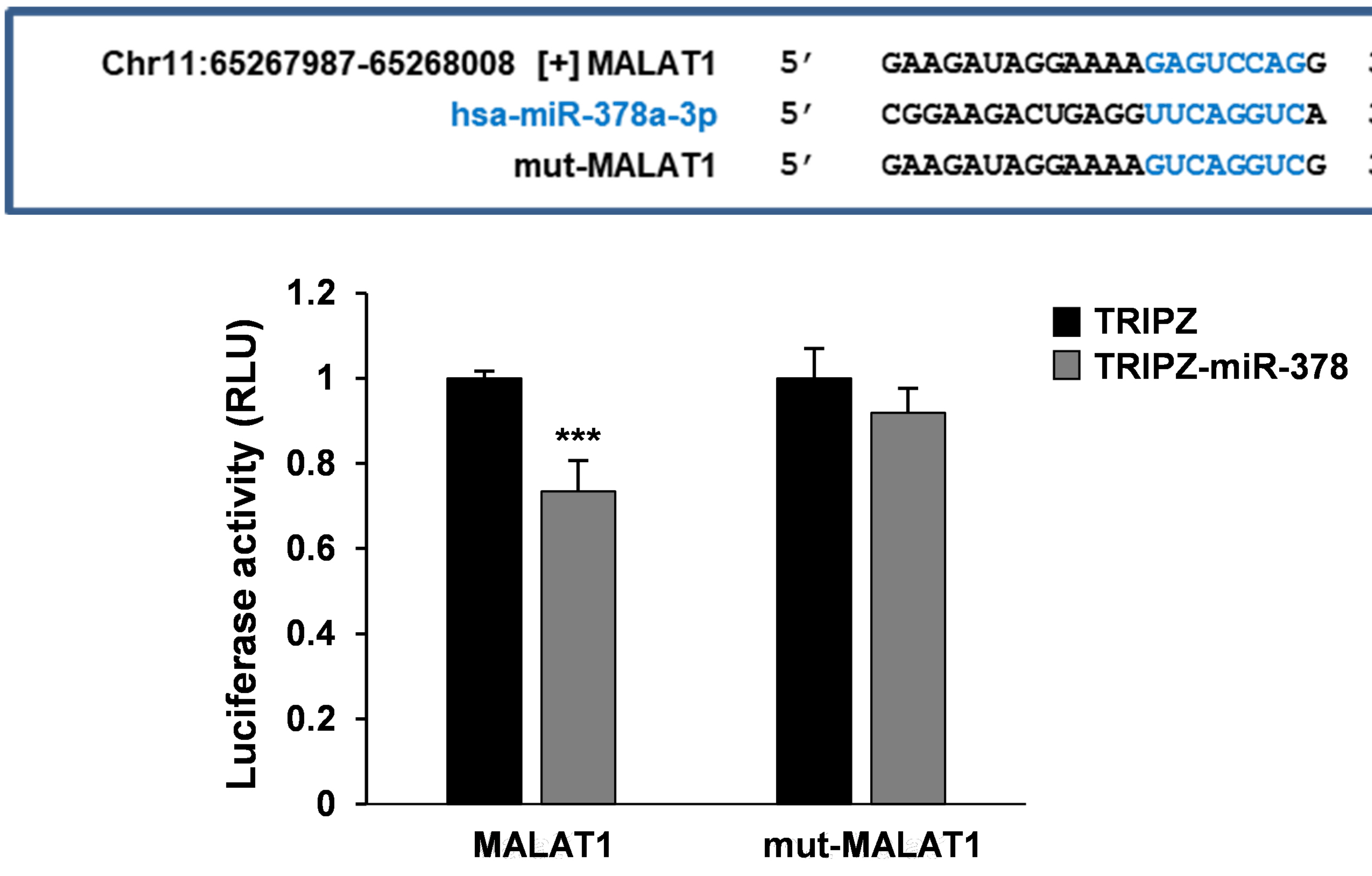

B

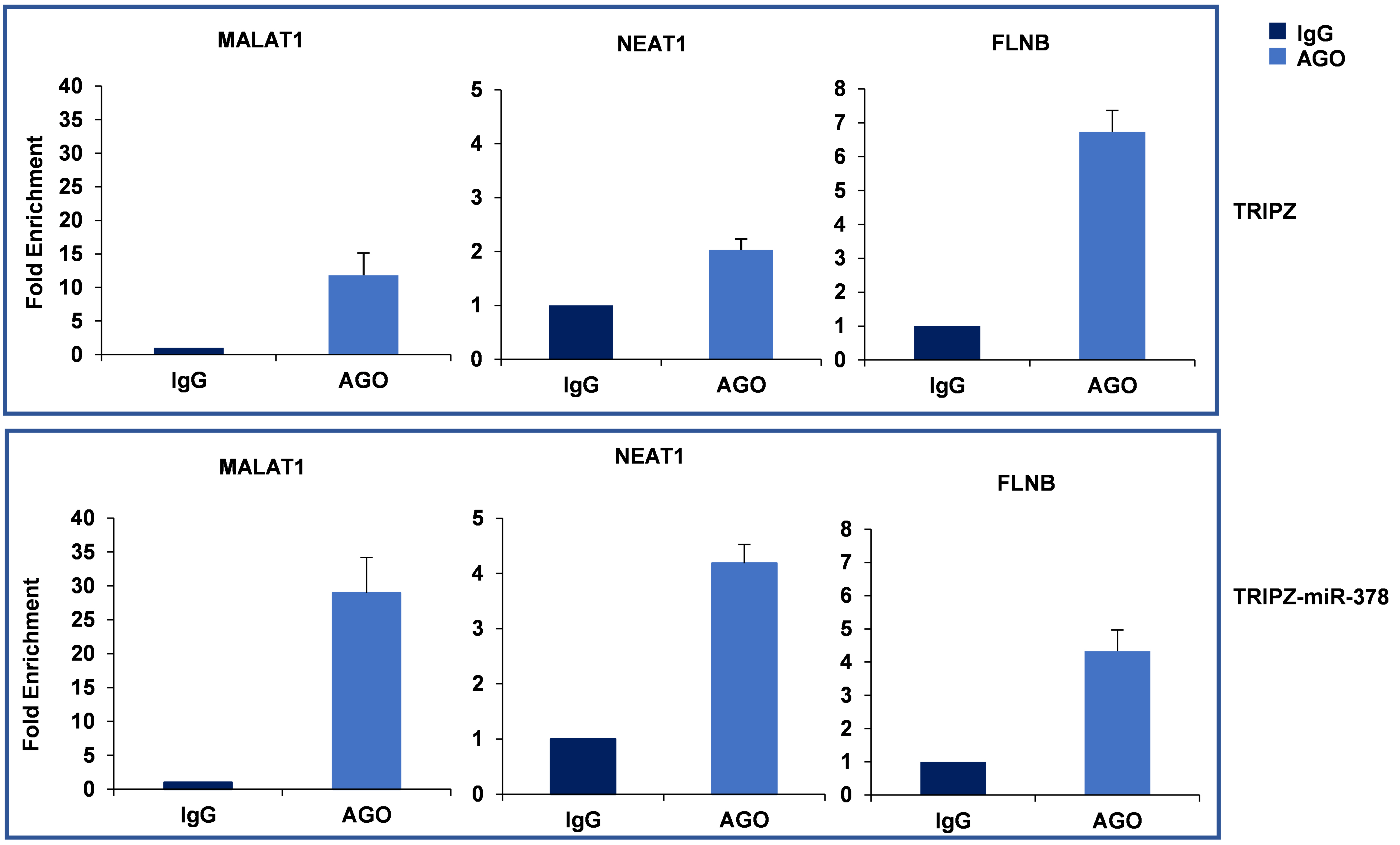

Supplementary Figure S2. A. Dual-luciferase reporter assay in DLD1 TRIPZ or TRIPZ-miR-378 transfected with reporter vectors containing the wild type (wt) or the mutated (mut) MALAT1 sequences. Histograms show normalized mean values of the relative luciferase activity. Error bars represent the mean ± SD (n = 3). ***, $p < 0.001$ based on Student's t test. **B.** qRT-PCR on RNA recovered after RNA Immunoprecipitation assay with a pan-Ago and control IgG antibodies to pulldown the endogenous MALAT1, NEAT1 and FLNB (miR-378a-3p non-target mRNA) in TRIPZ and TRIPZ-miR-378 DLD1 cells.

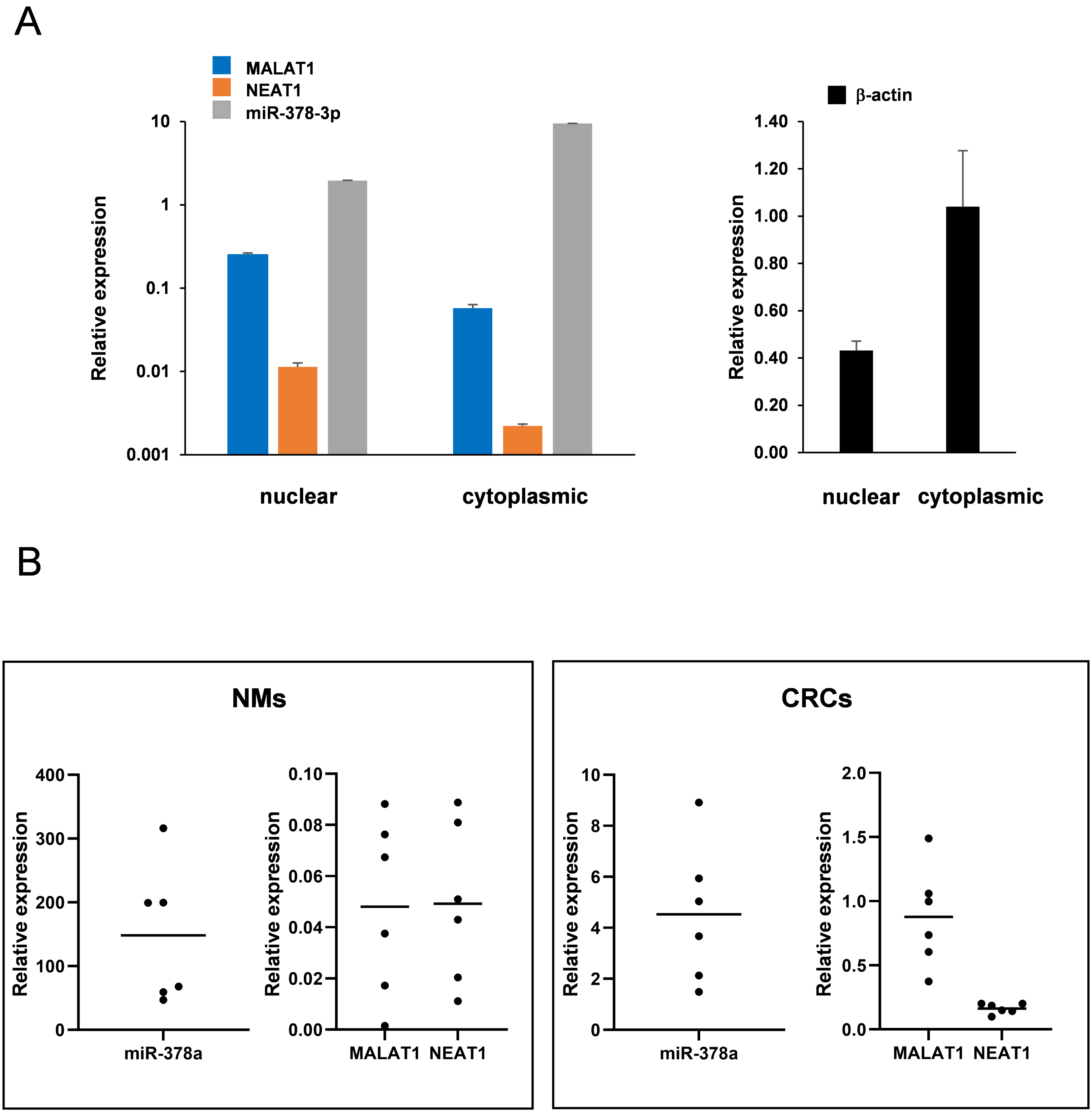

Supplementary Figure S3. A. qRT-PCR in nuclear and cytoplasmic fractions of RNA isolated from DLD1 cell line, showing that the three ncRNAs mainly co-localized in the nucleus. β-actin was used to validate the RNA fractionation. **B.** MiR-378a-3p, MALAT1 and NEAT1 expression was analyzed by qRT-PCR in normal mucosal tissues (NMs, n=6, *left panel*) and in CRC tissues (CRCs, n=6, *right panel*).
